# Preparation of Highly Catalytic N-Doped Carbon Dots and Their Application in SERS Sulfate Sensing

**DOI:** 10.3390/ma11091655

**Published:** 2018-09-07

**Authors:** Libing Wang, Chongning Li, Yanghe Luo, Zhiliang Jiang

**Affiliations:** 1 School of Food and Bioengineering, Hezhou University, Hezhou 542899, China; 18074841309@163.com (L.W.); lcn7882342@163.com (C.L.); 2 Key Laboratory of Ecology of Rare and Endangered Species and Environmental Protection (Guangxi Normal University), Ministry of Education, Guilin 541004, China; 3 Guangxi Key Laboratory of Environmental Pollution Control Theory and Technology, Guilin 541004, China

**Keywords:** N-doped carbon dots, catalysis, gold nanoreaction, SERS

## Abstract

Carbon dots (CD) have excellent stability and fluorescence activity, and have been widely used in fluorescence methods. However, there are no reports about using CD as catalysts to amplify SERS signals to detect trace sulfate. Thus, preparing CD catalysts and their application in SERS sulfate-sensing are significant. In this article, highly catalytic N-doped carbon dots (CD_N_) were prepared by a hydrothermal procedure. CD_N_ exhibited strong catalysis of the gold nanoparticle (AuNP) reaction between HAuCl_4_ and H_2_O_2_. Vitoria blue 4R (VB4R) has a strong SERS peak at 1614 cm^−1^ in the formed AuNP sol substrate. When Ba^2+^ ions were added, they were adsorbed on a CD_N_ surface to inhibit the CD_N_ catalytic activity that caused the SERS peak decreasing. Upon addition of analyte SO_4_^2−^, a reaction with Ba^2+^ produced stable BaSO_4_ precipitate and CD_N_, and its catalysis recovered to cause SERS intensity increasing linearly. Thus, an SERS method was developed for the detection of 0.02–1.7 μmol/L SO_4_^2−^, with a detection limit of 0.007 μmol/L.

## 1. Introduction

Because carbon dots (CD) have excellent stability, excellent chemical properties, high fluorescence activity, anti-photobleaching abilities and low cell toxicity [1,2,3,4,5], they are of interest to scientists. Based on the redox, complex, enzyme and immune reactions, CD have been used to determine chlorine ion, phosphate, ATP, ferric ion, hydrogen peroxide, glucose, immunoglobulin G, biological thiols, deoxyribonucleic acid, trypsin and so on [6,7,8,9,10,11]. Freire et al. [12] used polyvinyleneimine to prepare carbon quantum dots (CQDs/BPEI) to detect proteins. The nitrogen-doped carbon dots with high fluorescence efficiency have attracted much attention. Liu et al. [13] prepared nitrogen-doped graphene quantum dots and a photoelectrochemical aptasensor for chloramphenicol determination. Gu et al. [14] used 2-azidoimidazole and ammonia as reactants to prepare a fluorescent quantum dots by a thermal procedure, and to determine cysteine (Cys) by the reaction of CD-Cu^2+^-Cys. An aptamer has good electivity and has been combined with CD. Feng et al. [15] reported a graphene quantum dots-aptamer fluorescent probe to detect lead (II) ions (as low as 0.6 nmol/L). However, there are no reports about preparation of highly catalytic N-doped carbon dots and their application to SERS quantitative analysis.

SERS is a highly sensitive and selective molecular spectral technique; it has been used in biomedical, environmental monitoring, and analytical chemistry [16,17,18]. Liang et al. [19] prepared silver nanorods/reduced graphene oxide (AgNR/rGO) nanosol as SERS substrate to determine 8–1500 nmol/L iodide. Yang et al. [20] prepared silver nanosol SERS substrate to determine 2–191.0 mg/L thiocyanate. Luo et al. [21] prepared triangular nanosilver based on graphene oxide catalysis, and the nanosilver was used to analysis of 0.7–72 nmol/L nitrite by SERS. Jiang et al. [22] examined the catalytic reduction of HAuCl_4_ by cysteine with AuNP nanoenzyme to prepare gold nanosol substrate with high SERS activity to determine surfactants. Zhang et al. [23] developed a SERS method for detection of SO_2_, with a detection limit of 1 mg/L, based on the Raman peak at 630 cm^−1^ of S atom. Shang et al. [24] prepared silver nanochain (AgNC) sol substrate to analyze 0.00725–0.3 μmol/L hexametaphosphate. Sulfate is one of the important anions in water science, food science, soil chemistry, biology, mineralogy and related disciplines. For analysis of trace SO_4_^2−^, there are visible–ultraviolet spectrophotometry, turbidimetry, fluorescence spectrophotometry, electrochemical analysis, radiochemical analysis, resonance Rayleigh scattering, ion chromatography, and so on [25,26,27,28,29,30]. In this experiment, highly catalytic N-doped carbon dots were prepared for the HAuCl_4_-H_2_O_2_ reaction, and a new and sensitive SERS quantitative analysis method was proposed for the determination of sulfate in water and beer samples, based on the CD catalysis.

## 2. Materials and Methods

### 2.1. Apparatus and Reagents

The SERS spectra were recorded by a model of DXR smart Raman spectrometer (Thermo, Waltham, MA, USA) with laser wavelength of 633 nm, power of 3.5 mW, slit of 50 μm and acquisition time of 5 s. A model of 3K-15 high-speed refrigerated centrifuge (Sigma Co., Darmstadt, Germany) and a model of 79-1 magnetic stirrer with heating (Zhongda Instrumental Plant, Jiangsu, China) were used. A model of S-4800 field emission scanning electron microscope (Hitachi High-Technologies Corporation, Japan/Oxford Company, Oxford, UK) was used to record the graphs. 

A 2.9 mmol/L HAuCl_4_ (National Pharmaceutical Group Chemical Reagents Company, Shanghai, China), 10 μmol/L VB4R (Shanghai Reagent Three Factory, Shanghai, China) stock solution, 1 mmol/L BaCl_2_ (Hunan Reagent Factory, Changsha, China), 1.00 mmol/L Na_2_SO_4_ (Xilong Science Co., Ltd., Shantou, China) and 3.4 mmoL/L trisodium citrate (Xilong Chemical Plant, Shantou, China) were prepared.

Preparation of N-doped carbon dot solution (CD_N_): A 1 g of citric acid and 0, 0.5, 1.0 and 2.0 g urea were dissolved respectively in 30 mL water, and the brown yellow transparent solution was transferred to a polytetrafluoroethylene autoclave. After sealing, the autoclave was heated at 180 °C for 5 h. It was cooled to room temperature with tap water and was dialysis a night with dialysis bag of 3500 Da, and neutralized with NaOH solution to pH 7.0 to get a 0.021 g/mL CD_N_ that was named as CD_0N_, CD_0.5N_, CD_1N_ and CD_2N_ respectively.

### 2.2. Procedure

In a 5 mL graduated test tube, an appropriate amount of Na_2_SO_4_, 80 μL 1 mmol/L BaCl_2_ and 75 μL 100 μg/mL CD were added and mixed well. Then 100 μL 0.1% HAuCl_4_ and 50 μL 0.10 mol/L H_2_O_2_ solution were added and diluted to 1.5 mL. The tube was heated at 50 °C water bath for 20 min, cooled with ice-water, and 50 μL10 μmol/L VB4R molecular probe was added. The SERS spectrum was recorded by the spectrometer. The SERS peak intensity at 1614 cm^−1^ (*I*_1614cm^−1^_) and a blank (*I*_1614cm^−1^_)_0_ without SO_4_^2−^ were recorded. The value of Δ*I* = *I*_1614cm^−1^_ − (*I*_1614cm^−1^_)_0_ was obtained.

## 3. Results and Discussions

### 3.1. Principle

**Figure 1:**
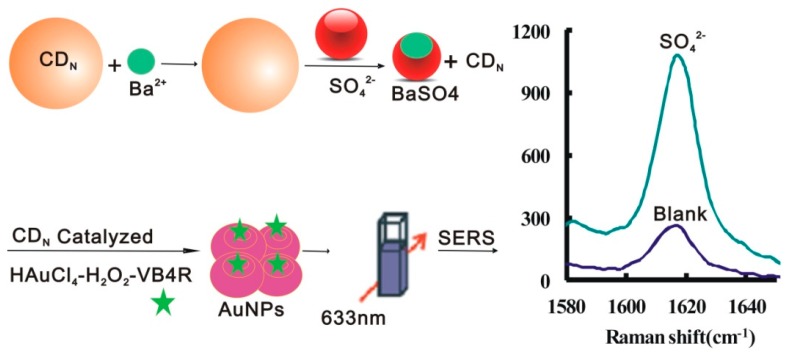
Surface enhanced Raman scattering (SERS) determination of sulfate by BaSO_4_ regulation of CD_N_ catalysis of the gold nanoreaction between HAuCl_4_ and H_2_O_2_.

The AuNP reaction was very slow, and the CD_1N_ surface contained more surface electrons that enhanced the electron transfer of the HAuCl_4_-H_2_O_2_ redox reaction, and displayed strong catalytic activity on the AuNP reaction. The Ba^2+^ ions adsorb on the CD_1N_ surface and repress the catalysis. When SO_4_^2−^ was added, stable BaSO_4_ formed and CD_1N_ was released which caused the SERS peak to increase due to formation of more SERS active gold nanoparticles. The more SO_4_^2−^ was added, the more CD was released, the more Au nanoparticles formed, and the SERS signal enhanced greatly after addition of probe VB4R. Accordingly, a new SERS quantitative analysis method was proposed for trace sulfate, based on the regulation of CD_1N_ catalysis (Figure 1).

### 3.2. SERS Spectra

**Figure 2:**
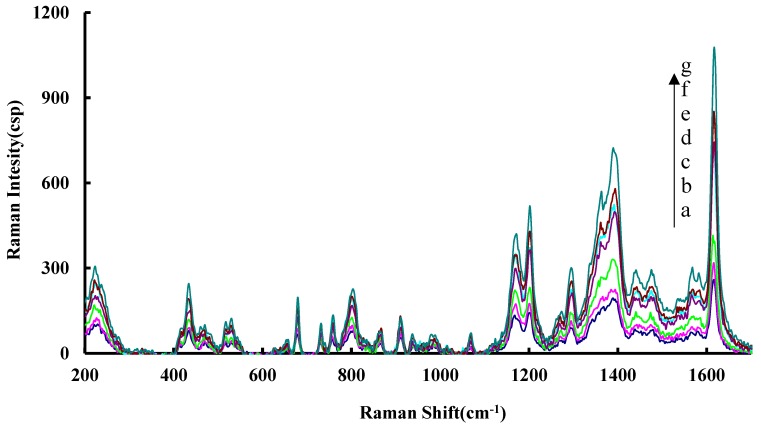
SERS spectrum of HAuCl_4_-H_2_O_2_-CD_1N_-Na_2_SO_4_-BaCl_2_-VB4R system. (**a**): 4.2 μmol/L HAuCl_4_ + 2.5 mol/L H_2_O_2_ + 5 μg/mL CD_1N_ + 53 μmol/L BaCl_2_ + 0.33 μmol/L VB4R; (**b**): a + 0.05 μmol/L Na_2_SO_4_; (**c**): a + 0.10 μmol/L Na_2_SO_4_; (**d**): a + 0.2 μmol/L Na_2_SO_4_; (**e**): a + 0.7 μmol/L Na_2_SO_4_; (f): a + 1.0 μmol/L Na_2_SO_4_; (**g**): a + 1.7 μmol/L Na_2_SO_4_.

Compared to common carbon nanomaterials such as graphene and C_60_, CD are very stable and dissolved in water, and were chosen for use. The CD_0N_, CD_0.5N_, CD_1N_ and CD_2N_ analytical systems were studied by an SERS technique with VB4R molecular probes. There are nine SERS peaks at 240, 432, 675, 800, 1175, 1202, 1290, 1394 and 1614 cm^−1^ (Figure 2). With the SO_4_^2−^ concentration increasing, the SERS signal increased greatly. Among the four systems, the CD_1N_ analytical system at 1614 cm^−1^ SERS peak is the most sensitive. Thus, it was chosen to detect SO_4_^2−^.

### 3.3. Scanning Electron Microscopy

**Figure 3:**
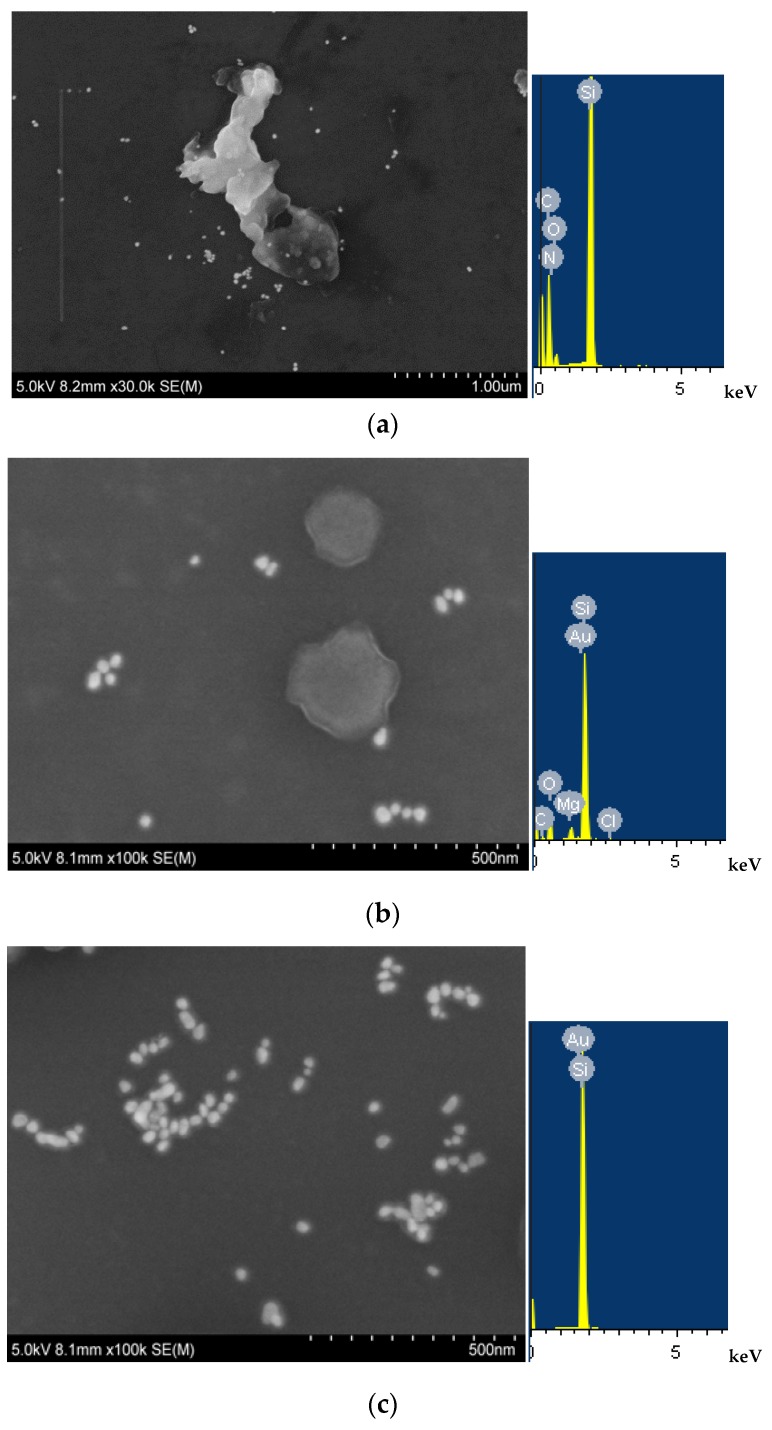
Scanning electron microscopy of the CD_1N_ analytical system. (**a**): 50 μg/mL CD_1N_; (**b**): 4.2 μmol/L HAuCl_4_ + 0.33 μmol/L VB4R + 5 μg/mL CD_1N_ + 2.5 mmol/L H_2_O_2_ + 53 mol/L BaCl_2_; (**c**): b + 1.67 μmol/L Na_2_SO_4_.

Scanning electron microscopy (SEM, Hitachi High-Technologies Corporation, Japan/Oxford Company, Oxford, UK) and energy spectra of CD_1N_ show that the small CD particles are spherical with an average size of 20 nm (Figure 3a) and the large aggregate may be the salt crystallization on the silicon wafer of SEM. There is a spectral peak at 0.25 keV for C, N and O elements. The SEM of HAuCl_4_-H_2_O_2_-CD_1N_-Na_2_SO_4_-BaCl_2_-VB4R was recorded. When there is no Na_2_SO_4_, the HAuCl_4_-H_2_O_2_ reaction is very slow to produce few quasi spherical AuNPs with an average size of 50 nm (Figure 3b); the morphology is not like the CD_1N_, and there is a spectral peak at 1.7 keV for Au. When Na_2_SO_4_ was added (Figure 3c), there were more AuNPs with an average size of 40 nm owing to CD_1N_ catalysis recovering and enhancing the SERS peak. This also indicated that the particles are AuNPs in the analytical system.

### 3.4. Catalysis and Inhibition

**Figure 4:**
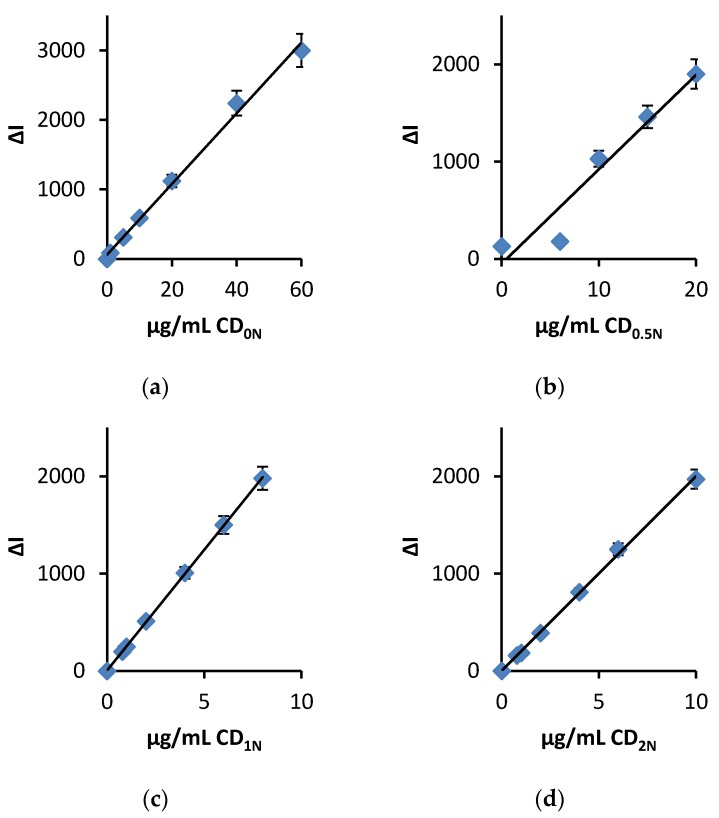
Relationship between the SERS intensity and CD catalyst concentration. 4.2 μmol/L HAuCl_4_ + 2.5 mmol/L H_2_O_2_ + CD_0N–2N_ + 0.33 μmol/L VB4R. (**a**) CD_0N_; (**b**) CD_0.5N_; (**c**) CD_1N_; (**d**) CD_2N_.

**Figure 5:**
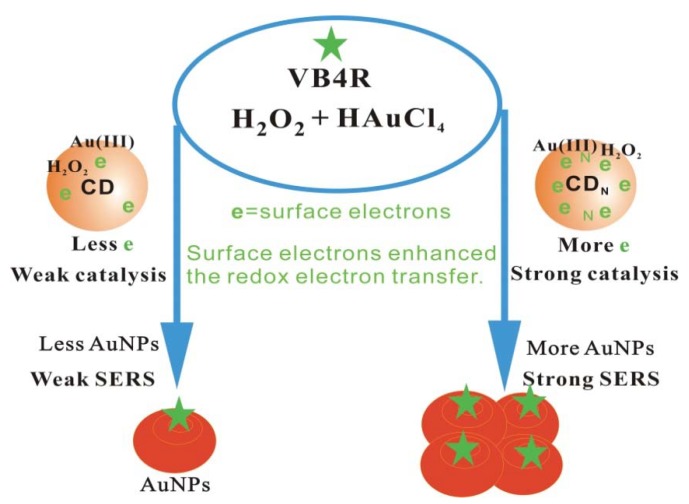
Enhancement of the doped N element.

Under the conditions as in the procedure, the AuNP reaction of H_2_O_2_-HAuCl_4_ is slow. The CDN exhibited catalysis of the AuNP reaction, and the SERS intensity increased with increasing CD concentration (Table 1, Figure 4). The CD without N element exhibited weak catalysis of the AuNP reaction of H_2_O_2_-HAuCl_4_, with a slope of 55.8. After doping N element such as CD_1N_ with a slope of 249, the CD_1N_ surface electrons were enhanced; the CD_1N_ surface electrons accelerated the redox electron transfer so that the gold nanoreaction was greatly enhanced to produce more AuNPs which caused the SERS intensity to increase (Figure 5).

### 3.5. Optimization of Analytical Conditions

**Figure 6:**
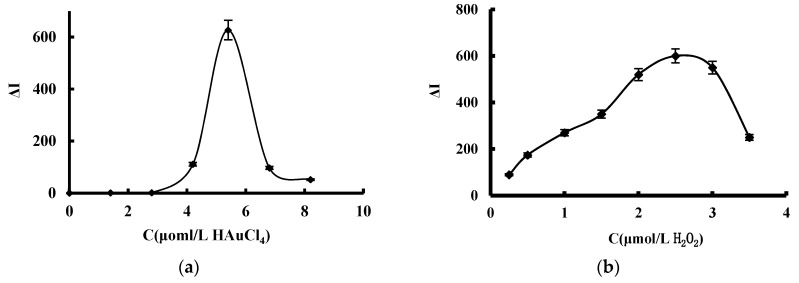

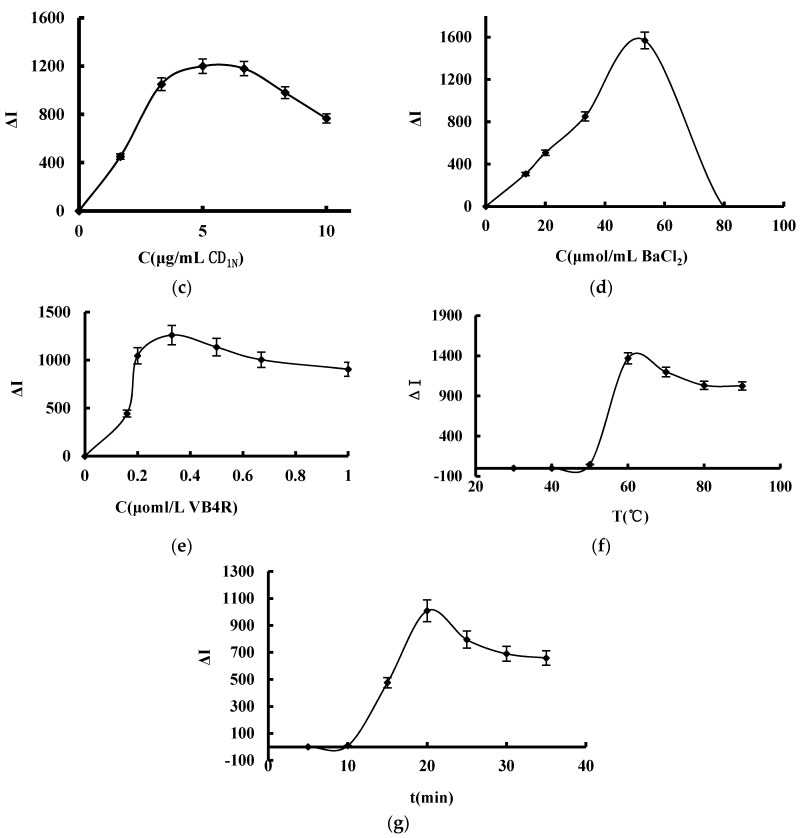
Effect of reagent concentration, reaction temperature and time. (**a**): HAuCl_4_ + 2.5 mmol/L H_2_O_2_ + 5 μg/mL CD_1N_ + 0.67 μmol/L Na_2_SO_4_ + 53 μmol/L BaCl_2_ + 0.33 μmol/L VB4R; (**b**): 4.2 μmol/L HAuCl_4_ + 0.33 μmol/L VB4R + 5 μg/mL CD_1N_ + 0.67 μmol/L Na_2_SO_4_ + 53 μmol/LBaCl_2_; (**c**): 4.2 μmol/L HAuCl_4_ + 2.5 mmol/L H_2_O_2_ + CD_1N_ + 0.67 μmol/L Na_2_SO_4_ + 53 μmol/L BaCl_2_ + 0.33 μmol/L VB4R; (**d**): 4.2 μmol/L HAuCl_4_ + 2.5 mmol/L H_2_O_2_ + 5 μg/mL CD_1N_ + 0.67 μmol/L Na_2_SO_4_ + BaCl_2_ + 0.33 μmol/L VB4R; (**e**): 4.2 μmol/L HAuCl_4_ + 2.5 mmol/L H_2_O_2_ + 5 μg/mL CD_1N_ + 0.67 μmol/L Na_2_SO_4_ + 53 μmol/L BaCl_2_ + VB4R; (**f**): Reaction temperature, 4.2 μmol/L HAuCl_4_ + 2.5 mmol/L H_2_O_2_ + 5 μg/mL CD_1N_ + 0.67 μmol/L Na_2_SO_4_ + 53 μmol/L BaCl_2_ + 0.33 μmoL/L VB4R; (**g**): Reaction time, 4.2 μmol/L HAuCl_4_ + 2.5 mmol/L H_2_O_2_ + 5 μg/mL CD_1N_ + 0.67 μmol/L Na_2_SO_4_ + 53 μmol/L BaCl_2_ + 0.33 μmoL/L VB4R.

The effect of reagent concentration such as HAuCl_4_, H_2_O_2_, CD_1N_, BaCl_2_ and VB4R, reaction temperature and time were optimized, respectively (Figure 6). When HAuCl_4_ is 4.2 μmol/L, most AuNPs formed in analytical systems with large Δ*I*. With increasing H_2_O_2_, the Δ*I* increased due to the formed AuNPs increasing, and a 2.5 mmol/L H_2_O_2_ gives the largest Δ*I*. CD_1N_ is the catalyst of the AuNP reaction, when the catalyst concentration increased, the Δ*I* enhanced, a 5 μg/mL CD_1N_ gives the largest Δ*I*. BaCl_2_ can inhibit the CD catalysis, when its concentration increased the Δ*I* was enhanced due to the blank decreasing, a 53 μmol/L BaCl_2_ gives the largest Δ*I*. VB4R is a sensitive molecular probe; when the concentration increased the Δ*I* enhanced due to more VB4R adsorption on the AuNP surface, a 0.33 μmoL/L VB4R gives biggest Δ*I*. Reaction temperature and time were considered; 50 °C for 20 min gives biggest Δ*I*. Thus, a 4.2 μmol/L HAuCl_4_, 2.5 mmol/L H_2_O_2_, 5 μg/mL CD_1N_, 53 μmol/L BaCl_2_ and 0.33 μmoL/L VB4R, and a reaction temperature of 50 °C for 20 min was selected in this SERS method. 

### 3.6. Performance Curve

**Figure 7:**
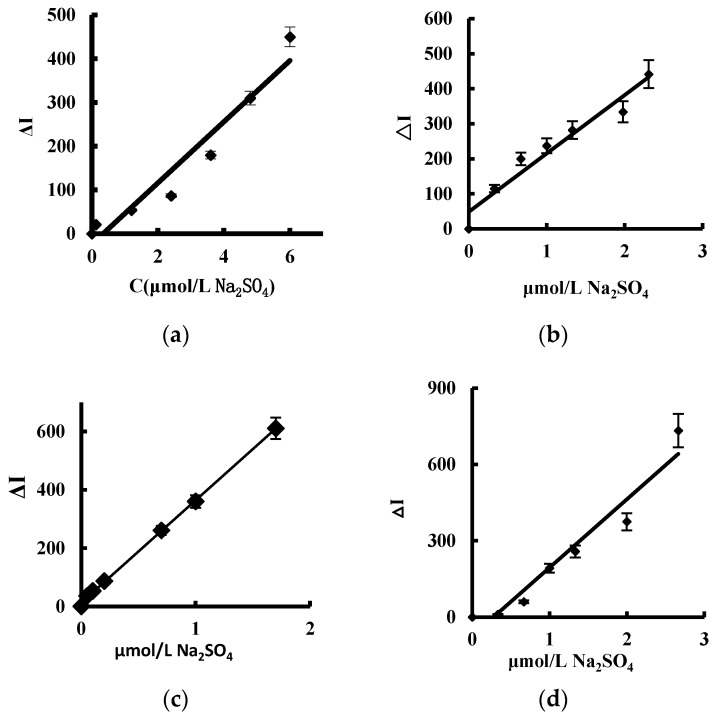
Working curves for CD catalytic-SERS method. 4.2 μmol/L HAuCl_4_ + 2.5 mmol/L H_2_O_2_ + 5 μg/mL CD + 53 μmol/L BaCl_2_ + 0.33 μmol/L VB4R. (**a**): CD_0N_; (**b**): CD_0.5N_; (**c**): CD_1N_; (**d**): CD_2N_.

The working curve of the system was drawn according to the experimental method. In the four systems (Table 2, Figure 7), the CD_1N_ was most sensitive, with a linear range (LR) of 0.02–1.7 μmol/L and a detection limit (DL) of 0.007 μmol/L, and was selected for detection of sulfate. Comparison of the reported methods for detection of sulfate [25,26,27,28,29,30] showed the SERS method was more sensitive. The C_60_ catalytic SERS method was used to detect sulfate, but the preparation of C_60_ is complex, the C_60_ nanosol is unstable [25], and the CD_1N_ analytical system overcomes the disadvantages.

### 3.7. Influence of Foreign Substances 

The influence of foreign substance on the determination of 0.66 µmol/L SO_4_^2−^ was investigated according to the experimental method. When the relative error is within 10%, results show that 33 µmol/L Na^+^, Zn^2+^, Ca^2+^, ethanol, Pb^+2^, NH_4_^+^, K^+^, SO_3_^2−^, Bi^3+^ and Cu^+2^, 26.4 µmol/L HCO_3_^−^, Mg^2+^, 16.5 µmol/L ethylene glycol, 6.6 µmol/L Cr^6+^, Fe^3+^, NO_2_^−^ and glycolic acid did not interfer with the SERS detection. Table 3 shows that the SERS quantitative analysis method has good selectivity.

### 3.8. Analysis of Samples

The water samples including tap water, Rong lake water and Shan lake water were taken into sample bottles. The lake water was filtered with filter paper, and then a 2.0 mL water sample was removed in a centrifuge tube. Three beer samples were purchased supermarkets. Samples were centrifuged at 7000 r/min for 10 min, to obtain a sample solution. The sulfate content was determined according to the SERS detection procedure. The SERS results were in agreement with that of ion chromatography (IC), the relative standard deviation was in the range of 0.90–4.77% and the recovery was between 92.3% and 105% (Table 4).

## 4. Conclusions

Highly catalytic CD_N_ was prepared by a hydrothermal procedure, and it was used to catalyze the reduction of chlorauric acid by H_2_O_2_ to produce AuNP sol substrate with high SERS activity. Ba(II) ions can combined with CD_N_ to inhibit the catalysis of CD_N_. Upon addition of sulfate ions, stable barium sulfate precipitates formed, and CD_N_ was released, which causes CD_N_ catalysis to be activated and the SERS signal to be enhanced. Based on this principle, a new, simple and selective SERS quantitative analysis method was established for the detection of trace sulfate. 

## Figures and Tables

**Table 1:** Comparing of the catalysis by SERS method *^a^*.

System	Linear Range	Regress Equation	Coefficient
CD_0N_	1.0–60 μg/mL	Δ*I* = 55.8x + 30	0.9898
CD_0.5N_	6.0–20 μg/mL	Δ*I* = 89.2x + 130	0.9869
CD_1N_	0.79–8 μg/mL	Δ*I* = 249.0x − 8.6	0.993
CD_2N_	0.79–10 μg/mL	Δ*I* = 197.4x + 13	0.9633

**Table 2:** Analytical features of CD-catalytic SERS determination of sulfate.

CD	Linear Equation	Coefficient	LR (μmol/L)	DL(μmol/L)
CD_0N_	Δ*I* = 66.9C + 20.4	0.9283	1.0–6.0	0.50
CD_0.5N_	Δ*I* = 166.4C + 48.8	0.9463	0.5–2.31	0.20
CD_1N_	Δ*I* = 348.8C + 18.0	0.9384	0.02–1.7	0.007
CD_2N_	Δ*I* = 268.6C−73.9	0.9403	0.06–2.66	0.02

**Table 3:** Effect of interfering substances on the SERS detection of 0.66 µmol/L SO_4_^2−^.

Foreign Substance	Tolerance Concentration (µmol/L)	Relative Error (%)	Foreign Substance	Tolerance Concentration (µmol/L)	Relative Error (%)
Na^+^	33	5.0	Cu^2+^	33	7.6
Zn^2+^	33	6.4	HCO_3_^−^	26.4	8.6
Ca^2+^	33	−6.7	Mg^2+^	26.4	6.0
ethanol	33	−5.6	ethylene glycol	16.5	5.8
Pb^2+^	33	7.0	Cr^6+^	6.6	−6.0
NH_4_^+^	33	3.9	Fe^3+^	6.6	−4.5
K^+^	33	6.0	NO_2_^−^	6.6	6.2
SO_3_^2−^	33	−7.9	glycolic acid	6.6	5.0
Bi^3+^	33	6.4			

**Table 4:** Analytical results of sulfate in water samples.

Sample	Single Value (μmol/L)	Average (μmol/L)	Added (μmol/)	Found (μmol/L)	Recovery (%)	RSD (%)	Content (μmol/L)	IC Results (μmol/L)
Running water	0.39, 0.41, 0.38, 0.40, 0.43	0.40	0.13	0.52	92.3	4.77	0.40	0.38
Ronng lake water	1.12, 1.17, 1.11, 1.17, 1.17	1.15	0.13	1.274	95	2.6	1.15	1.22
Shan lake water	0.70, 0.71, 0.71, 0.72, 0.71	0.71	0.13	0.839	99.2	0.90	0.71	0.68
Beer 1	1.22, 1.26, 1.30, 1.28, 1.32	1.28	0.20	1.47	95	3.0	1.28	1.20
Beer 2	1.30, 1.35, 1.38, 1.39, 1.33	1.35	0.20	1.56	105	2.7	1.35	1.38
Beer 3	1.39, 1.30, 1.39, 1.32, 1.25	1.33	0.20	1.52	95	4.3	1.33	1.28
